# An Incidental Diagnosis of Neurosyphilis: A Case Report

**DOI:** 10.7759/cureus.49299

**Published:** 2023-11-23

**Authors:** Madeline Franke, Tanner Walker, Yahor A Sukharutski, Christopher R Force

**Affiliations:** 1 Internal Medicine, Texas A&M College of Medicine, Houston, USA; 2 Internal Medicine, Houston Methodist Hospital, Houston, USA

**Keywords:** syphilis, case report, infectious disease, neurology, treponema pallidum, optic neuropathy, neurosyphilis

## Abstract

Neurosyphilis, a rare manifestation of *Treponema pallidum* spreading into the central nervous system, is a critical differential diagnosis due to its potentially severe neurologic effects if left untreated. We present a case of a 37-year-old woman who complained of bilateral vision loss and headaches originally concerning for idiopathic intracranial hypertension and uncontrolled diabetes. Comprehensive evaluations eventually led to a neurosyphilis diagnosis. Ophthalmologic examination revealed multifactorial visual symptoms with diabetic retinopathy contributing alongside neurosyphilis. Treatment was started promptly at the time of diagnosis and resulted in improvement in visual symptoms. This case emphasizes the importance of syphilis screening in appropriate age ranges, even in low-prevalence areas. Recognition of neurosyphilis as a potential diagnosis can impact patient outcomes and highlights the need for continued vigilance to identify the disease.

## Introduction

Syphilis, characteristically caused by the spirochete bacterium *Treponema pallidum*, is a systemic infection more common in developing countries [1]. Neurosyphilis represents the advanced stage of the disease when the bacteria begins to invade the central nervous system. Despite being relatively rare in the developed world since the advent of penicillin treatment, it remains a critical differential due to its potentially irreversible sequelae. Early recognition with screening venereal disease research laboratory (VDRL) testing and appropriate treatment is imperative in patients with suspected neurosyphilis to prevent long-term neurological deficits. In some asymptomatic or atypical populations, screening might still be appropriate. For instance, it has been demonstrated that HIV-1-positive individuals can present with an increased incidence of asymptomatic neurosyphilis [2]. In this report, we present a case of a 37-year-old woman presenting with complaints of headaches and bilateral vision loss whose progression to neurosyphilis was not initially detected.

## Case presentation

History of present illness

A 37-year-old Hispanic woman with a history of hypertension, insulin-dependent diabetes mellitus, and class 2 obesity with a body mass index (BMI) of 35 kg/m^2^ presented to the hospital with a two-month history of bilateral vision loss and headaches. The patient reported that her visual symptoms and headache both initially began approximately 17 months prior to presentation. At that time, she saw her optometrist, who gave her prescription eyeglasses and noted that she had optic disc edema on the exam. She did not seek further evaluation until two months later at her follow-up optometry visit, where she was instructed to go to the emergency department (ED) after she was found to have persistent bilateral optic disk edema and new bilateral retinal hemorrhages on the fundoscopic exam. She described her visual changes as progressive in nature, starting out as bilateral “shadows” blocking her visual fields with increased difficulty in differentiating between colors. This bilateral vision loss continued to develop until she was unable to drive safely during the day and night. On presentation to the hospital, the patient had bilateral swollen eyes and “gray, foggy” vision.

In addition to her visual loss, she also experienced a persistent frontal headache with associated bilateral, pulsatile tinnitus during this time. Her headache was reported as five out of 10 in severity, worsened by certain position changes, such as lying down. She denied any recent fever, eye pain, photo/phonophobia, chills, nausea, vomiting, diarrhea, recent weight gain, focal neurological weakness, or neck pain. She was not taking any new medications, had no oral contraceptive use, and denied any history of blood clots.

The patient denied any current alcohol or tobacco use. She occasionally used marijuana. She was sexually active with men only, reported using protection consistently, and denied any history of sexually transmitted infections or other notable sexual behavior. Of note, she did not have any previous screenings for sexually transmitted diseases on record. Her family history was noncontributory.

Clinical findings and diagnostic assessment

When the patient first arrived to the ED, she was afebrile, tachycardic with a heart rate of 101 beats per minute, and hypertensive at 154/88 mmHg. On physical exam, she was not in acute distress but had confirmed optic disk edema in both eyes along with decreased visual acuity. She did not have any nystagmus, extraocular muscle deficits, facial asymmetry, eye discharge, or conjunctival changes. She had no cranial nerve deficits, focal weakness, or other neurological symptoms. A follow-up physical exam demonstrated a small fiber sensory polyneuropathy in the upper and lower extremities with decreased reflexes in the bilateral lower extremities. Laboratory evaluation showed an elevated blood glucose of 412 mg/dl without evidence of diabetic ketoacidosis. Her complete blood count was notable for microcytic anemia and mild thrombocytosis. A COVID-19 polymerase chain reaction test was negative. A computed tomography (CT) scan with contrast of her orbits was performed, which did not show any appreciable hemorrhage or acute abnormality of the globes. She was admitted to the hospital for further workup and evaluation by neurology and ophthalmology.

**Figure 1:**
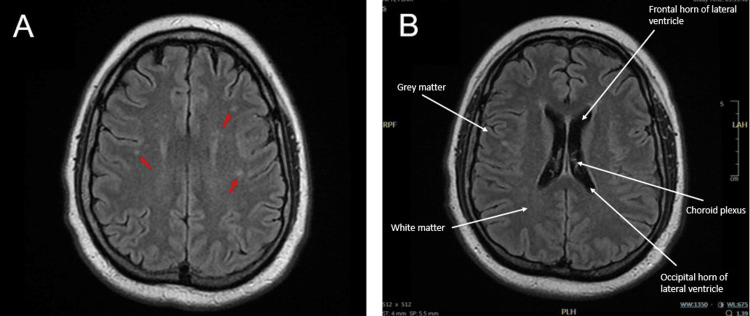
Axial T2 FLAIR MRI images of the brain and orbits revealed minimal white matter lesions characteristic of demyelinating disease. Figure A demonstrates some subcortical white matter hyperintensity (red arrows), but is otherwise unremarkable. Like Figure A, Figure B shows an axial cross-section of the brain with no evidence of hydrocephalus or other acute intracranial abnormality. FLAIR: fluid-attenuated inversion recovery.

Further evaluation with a magnetic resonance image (MRI) venogram was unremarkable for any acute intracranial abnormalities, significant stenosis, or thrombosis. A follow-up MRI of the brain and orbits revealed scattered subcortical and juxtacortical white matter lesions characteristic of demyelinating disease (Figure 1). Additionally, some periventricular white matter changes were present. An MRI focused on the patient’s cervical and thoracic spine was unrevealing for any demyelinating lesions. No other causes for her visual disturbance and headache were noted on imaging.

Ophthalmology was consulted for evaluation and found normal intraocular pressure, mild optic disc edema bilaterally, and macular and peripheral flame hemorrhage and cotton wool spots. Early serological studies for infectious sources were negative, including *Cytomegalovirus*, Epstein-Barr virus, herpes simplex virus, varicella-zoster virus, *Cryptococcus*, West Nile virus, mycobacteria, and HIV. An autoimmune workup was performed to test for the presence of diseases such as lupus, Sjogren's, systematic scleroderma, and rheumatoid arthritis. This workup was widely unremarkable with negative antinuclear antibodies (ANA), anti-cyclic citrullinated peptide (anti-CCP) antibodies, anti-double-stranded DNA (anti-dsDNA) antibodies, and anti-Scl-70 antibodies.

The syphilis antibody was positive with a subsequent rapid plasma reagin (RPR) titer of 1:256. A lumbar puncture revealed a normal opening pressure of 17 cmH2O and CSF with elevated glucose of 116 mg/dl, white blood cell count of 7 per µL, red blood cell count of 0 per µL, and a normal protein of 29 mg/dl. A VDRL CSF test was performed and was reactive.

A fluorescein angiogram was performed by ophthalmology, which revealed neovascularization of the disc with microaneurysms, peripapillary leakage, and blockage from intraretinal hemorrhages. No other acute abnormalities were found contributing to the patient’s visual deficits.

Therapeutic intervention

The patient was promptly treated with a 14-day regimen of intravenous penicillin G at a dosage of 4.0 million units every four hours. This was started in the hospital and continued outpatient through a peripherally inserted central catheter line. Her visual symptoms were found to be multifactorial due to her uncontrolled diabetes (proliferative diabetic retinopathy, 11.6 glycosylated hemoglobin) and a new diagnosis of neurosyphilis (bilateral optic neuropathy secondary to neurosyphilis). Her vision improved following the antibiotic course.

Follow-up and outcomes

The patient had follow-up visits with infectious disease and ophthalmology. It is unclear exactly when she contracted syphilis, but the patient later recalled a possible exposure five years prior to presentation.

Ophthalmology recommended pan-retinal photocoagulation of her right eye diabetic retinopathy for further management, and she has since undergone multiple treatments. Since discharge, the patient reported a resolution of her headache and tinnitus but is still experiencing visual symptoms. She is currently being followed up by ophthalmology for management of her diabetic retinopathy with interval improvement in optic disc edema and visual fields since completion of syphilis therapy.

The patient was ultimately diagnosed with neurosyphilis and associated bilateral optic neuropathy. This diagnosis was confirmed through reactive CSF VDRL, RPR, and syphilis total antibody as well as therapeutic response to antibiotic treatment. Her vision deficit has experienced incomplete recovery given the underlying diabetic retinopathy.

## Discussion

Our case follows a 37-year-old Hispanic woman presenting with a two-month history of bilateral vision loss and headaches who was eventually found to have neurosyphilis. Since the introduction of penicillin, neurosyphilis has become uncommon, affecting approximately 0.47 to 2.1 cases per 100,000 people [3]. We present a case of neurosyphilis that went undetected for likely over a year and presented atypically in a young, healthy patient. Our case emphasizes the importance of keeping neurosyphilis as a possible diagnosis since it can present in a variety of ways even years after exposure. As a disease with many adverse effects yet a simple treatment, the most critical part of the diagnosis is recognizing it as part of the differential and ordering the appropriate laboratory studies. Our report highlights neurosyphilis as a disease that is often overlooked due to its many manifestations and overall rarity [3].

Syphilis is usually transmitted between individuals through sexual contact [3]. Syphilis has different stages of infection, ranging from primary syphilis to tertiary syphilis, with different characteristic signs and symptoms [3]. Notably, syphilis has a latent phase between the secondary and tertiary stages where the disease can go dormant in an infected individual for years before it progresses [4]. While rates have previously been stable, more recent trends show a fairly rapid increase in prevalence with a near doubling of cases from 2017 to 2021 [5]. Neurosyphilis, in particular, refers to the disease at any stage when it spreads to the central nervous system. There are many forms of neurosyphilis, depending on how it presents in the patient: early neurosyphilis, late neurosyphilis, and atypical neurosyphilis. Early neurosyphilis includes manifestations such as asymptomatic neurosyphilis, symptomatic meningitis, ocular syphilis, and meningovascular syphilis [3]. Late neurosyphilis involves more severe forms of the disease, such as general paresis and tabes dorsalis [3,6]. Laboratory diagnosis of neurosyphilis depends on serologic tests (VDRL and RPR) in the serum and CSF [3,7].

Direct microbiological detection of *Treponema pallidum* is not routine since the bacteria cannot be cultured [7]. Our patient had positive CSF VDRL and serum RPR, which have sensitivities of 75% and 100% in early neurosyphilis, respectively [3]. Likewise, the specificities for positive CSF VDRL and serum RPR are 100% and 90%, respectively [3]. As the disease transitions from early neurosyphilis to late neurosyphilis (paresis and tabes dorsalis), sensitivity and specificity for these tests all decrease [3]. CSF content (white blood cell count, protein level) is also important in the diagnosis of neurosyphilis, with a white blood cell count > 5-10/mm3 and protein > 45 mg/dl, but these results alone cannot establish a diagnosis of neurosyphilis.

Our patient likely presented either with asymptomatic neurosyphilis (in the setting of diabetic retinopathy) or ocular neurosyphilis. It is unclear which manifestation was more predominant at the time of diagnosis. In either instance, our patient’s neurosyphilis was likely in the early stage rather than the late stage. There have been many case reports of early-stage neurosyphilis, all with different presenting symptoms and clinical courses [8]. For ocular neurosyphilis, any part of the eye can be involved. Commonly, inflammatory processes like posterior uveitis and panuveitis are noted upon diagnosis [9]. These conditions lead to loss of visual acuity and other complications such as glaucoma, cataracts, macular edema, retinal detachment, retinal vessel occlusion, and choroidal neovascularization [9]. Many patients with ocular involvement do not show any evidence of disease in their imaging, which can lead to difficulty in diagnosis [10].

The differential diagnosis for our case was broad, including various neurological and ophthalmological conditions. Since our patient presented with a headache and persistent optic disk edema, idiopathic intracranial hypertension was a leading concern. Idiopathic intracranial hypertension is a multifaceted neuro-ophthalmic condition highly prevalent in women of reproductive age who are overweight or obese [11]. It can cause rapid vision loss on presentation, so it was of high priority to rule out this disorder quickly [11]. Other diagnoses on the differential included inflammatory, infectious, and demyelinating diseases of the central nervous system, hypertension retinopathy, diabetic retinopathy, systemic inflammatory or infectious processes, and autoimmune conditions. The lumbar puncture revealed a normal opening pressure in our patient, essentially ruling out idiopathic intracranial hypertension. All of the imaging performed was widely unremarkable for the cause of our patient’s visual symptoms and headache. Eventually, the serology for syphilis was reactive in the CSF and serum, which finally established the diagnosis of neurosyphilis in our patient.

In our case, further workup by ophthalmology revealed that the cause of her visual symptoms was multifactorial. This was due to the physical exam findings and further testing that ophthalmology performed on an outpatient basis and the fact that her visual symptoms did not fully resolve once the neurosyphilis was treated. This is significant because there is a chance that her neurosyphilis diagnosis would have been missed if it were not for her progressive visual disturbances. The entire reason she presented to the ED initially was due to the advice of her optometrist after noting persistent optic disk edema and new retinal hemorrhages. While retinal hemorrhages were likely secondary to her diabetic eye disease, further evaluation ultimately uncovered the neurosyphilis diagnosis. It is important to always consider neurosyphilis as a possibility in complex neuro-ophthalmic cases due to the consequence of delayed treatment. Prompt diagnosis is essential since this disease can cause permanent neurological deficits and is easily treated with a short course of penicillin G [8].

## Conclusions

Our case highlights the importance of considering neurosyphilis on the differential in patients with bilateral vision loss in sexually active age ranges, even in regions such as the United States, where the diagnosis is rare but rapidly growing in prevalence. The presentation can be atypical and varied, requiring a high index of suspicion from clinicians. Early recognition and treatment can prevent severe long-term neurologic consequences with a readily available penicillin G course. This case serves as a reminder to healthcare professionals of the enduring significance of syphilis screening and its potentially disastrous consequences if left unchecked.

## References

[1] Schmidt RP (1992). Neurosyphilis. Clinical Neurology.

[2] Berger JR (1991). Neurosyphilis in human immunodeficiency virus type 1-seropositive individuals. A prospective study. Arch Neurol.

[3] Ropper AH (2019). Neurosyphilis. N Engl J Med.

[4] Mattei PL, Beachkofsky TM, Gilson RT, Wisco OJ (2012). Syphilis: a reemerging infection. Am Fam Physician.

[5] (2023). Centers for Disease Control and Prevention. Sexually transmitted disease surveillance, 2021. http://www.cdc.gov/std/statistics/2021/default.htm.

[6] Jantzen SU, Ferrea S, Langebner T, Gaebel W, Griese M, Arendt G, Dihné M (2012). Late-stage neurosyphilis presenting with severe neuropsychiatric deficits: diagnosis, therapy, and course of three patients. J Neurol.

[7] Tuddenham S, Katz SS, Ghanem KG (2020). Syphilis laboratory guidelines: performance characteristics of nontreponemal antibody tests. Clin Infect Dis.

[8] Kopp I (1948). Early neurosyphilis. N Engl J Med.

[9] Singh AE (2020). Ocular and neurosyphilis: epidemiology and approach to management. Curr Opin Infect Dis.

[10] Zhou X, Peng S, Song T, Tie D, Tao X, Jiang L, Zhang J (2022). Neurosyphilis with ocular involvement and normal magnetic resonance imaging results affirmed by metagenomic next-generation sequencing. Front Cell Infect Microbiol.

[11] Wang MT, Bhatti MT, Danesh-Meyer HV (2022). Idiopathic intracranial hypertension: pathophysiology, diagnosis and management. J Clin Neurosci.

